# KmerKeys: a web resource for searching indexed genome assemblies and variants

**DOI:** 10.1093/nar/gkac266

**Published:** 2022-04-26

**Authors:** Dmitri S Pavlichin, HoJoon Lee, Stephanie U Greer, Susan M Grimes, Tsachy Weissman, Hanlee P Ji

**Affiliations:** Division of Oncology, Department of Medicine, Stanford University School of Medicine, Stanford, CA, 94305, USA; Division of Oncology, Department of Medicine, Stanford University School of Medicine, Stanford, CA, 94305, USA; Division of Oncology, Department of Medicine, Stanford University School of Medicine, Stanford, CA, 94305, USA; Stanford Genome Technology Center West, Stanford University, Palo Alto, CA, 94304, USA; Department of Electrical Engineering, Stanford University, Palo Alto, CA, 94304, USA; Division of Oncology, Department of Medicine, Stanford University School of Medicine, Stanford, CA, 94305, USA; Stanford Genome Technology Center West, Stanford University, Palo Alto, CA, 94304, USA

## Abstract

K-mers are short DNA sequences that are used for genome sequence analysis. Applications that use k-mers include genome assembly and alignment. However, the wider bioinformatic use of these short sequences has challenges related to the massive scale of genomic sequence data. A single human genome assembly has billions of k-mers. As a result, the computational requirements for analyzing k-mer information is enormous, particularly when involving complete genome assemblies. To address these issues, we developed a new indexing data structure based on a hash table tuned for the lookup of short sequence keys. This web application, referred to as KmerKeys, provides performant, rapid query speeds for cloud computation on genome assemblies. We enable fuzzy as well as exact sequence searches of assemblies. To enable robust and speedy performance, the website implements cache-friendly hash tables, memory mapping and massive parallel processing. Our method employs a scalable and efficient data structure that can be used to jointly index and search a large collection of human genome assembly information. One can include variant databases and their associated metadata such as the gnomAD population variant catalogue. This feature enables the incorporation of future genomic information into sequencing analysis. KmerKeys is freely accessible at https://kmerkeys.dgi-stanford.org.

## INTRODUCTION

Large genomic sequencing projects have been generating a wealth of variants in the population. Citing an example, catalogues of variants derived from population studies include the Genome Aggregation Database **(gnomAD)** (1), ClinVar (2) and The Cancer Genome Atlas **(TCGA)**. These studies are broadening our understanding of the full range of human genetic diversity and facilitate the discovery of genetic factors that influence disease susceptibility (3,4). The interpretation of variants requires links to the human reference genome. However, the current reference was constructed using the genome sequence of a small number of individuals and as a result, does not account for many genomic features across the breadth of human genetic diversity (5–7). Addressing this limitation, there are ongoing projects that involve constructing a pangenome reference derived from a broader sampling of the human population (8,9). These aggregated reference assemblies from hundreds of individuals would improve the sequence analysis (8,10). Linking variant catalogues to this next generation of human reference genomes at this scale poses a significant challenge, which limits the accessibility for genomic researchers. Therefore, annotation of genomic features requires a format that can be related to different genome assemblies.

We developed a method for using K-mers for genomic annotation that includes genomic coordinates, counts, and pointers to datasets. K-mers are nucleotide sequences of length K. K-mer analysis methods are appealing in their conceptual simplicity, because these short sequences can be readily manipulated and compared among different sequence data sets. K-mer-based tools have a variety of different functions that include: enumeration (11,12), read filtering (13), evolutionary distance estimation (14), metagenomics (15), and RNAseq analysis (16). The majority of applications are geared towards mapping sequences from FASTA/Q files. Beyond mapping, k-mers have specific advantages for organizing and querying sequence databases; one can index genomic data, facilitate the organization of these data sets and offer highly efficient querying of large collections of genomic sequence data. Along these lines, we developed a website data resource that indexes k-mer sequences for reference assemblies and links them to variant catalogues from gnomAD.

## MATERIALS AND METHODS

### Overview of KmerKeys

**Figure 1.**
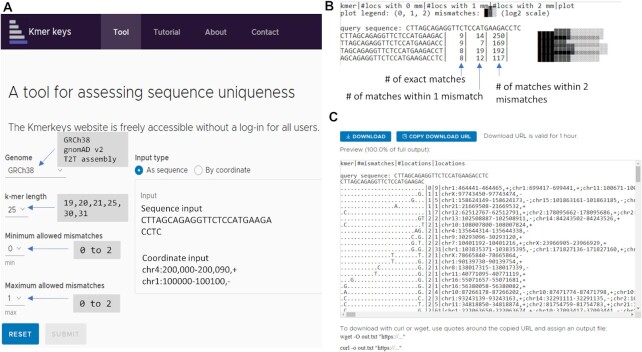
(**A**) Web application of KmerKeys. There are three indices available to query; i) GRCh38, ii) T2T assembly of CHM13, and iii) gnomAD v2. Users can query these indices based on k-mer length, and using either sequences or coordinates as input. Results will be generated with (**B**) The summary output shows the overall frequency of all query k-mers grouped by edit distance in the following format: i) the sequence of the k-mer, ii) the number of locations with an exact match, iii) the number of locations with an edit distance of 1 and iv) the number of locations with an edit distance of 2 the user's choice of fuzzy search by setting minimum/maximum allowed mismatches. (**C**) The detailed output shows: i) the frequency of a given sequence at a specific assembly coordinate, ii) the number of neighbor k-mers that are a small edit distance away, iii) the frequency of neighbor k-mers with their locations, and iv) the positions of the mismatching nucleotides on the neighbor k-mers.

KmerKeys is our web portal (https://kmerkeys.dgi-stanford.org/) deployed as a public cloud-hosted service on Amazon Web Services **(AWS)**. KmerKeys has the following cloud-based indices: ∼2.5 billion distinct 31-mers from two genome assemblies (GRCh38 and a T2T assembly of CHM13) and 17 million exonic variants from gnomAD (version 2.1.1) (Figure 1A). We used six different lengths of k-mers; 19, 20, 21, 25, 30 and 31. All indices on KmerKeys are searchable either by sequence in FASTA-style input or by a set of intervals within a selected dataset in genomic coordinates. When querying by sequence, an input string is decomposed into its constituent k-mers in a sliding window fashion, which are then individually queried against the hash table. When querying by coordinate, we first retrieve the sequence at the specified coordinates and then query its constituent short k-mers. Both exact and fuzzy queries are supported, the latter performed up to two mismatches.

The KmerKeys website was designed and optimized for high query speed, anticipating that indices would be rarely constructed and frequently queried. This choice of trade-off is suited for a cloud-based shared resource where the same memory and compute resources are shared by multiple users, and the architecture can straightforwardly scale to ever more assemblies and larger datasets. All visitors’ queries to the KmerKeys public resource use the same pool of memory and threads, thus reducing the average cost per query. The web application also allows anyone without a computational background to readily access our tool.

### Inputs and outputs

KmerKeys takes input either as sequence in FASTA-style or a set of intervals within a selected dataset in genomic coordinates. There are two types of outputs (Figure 1B and C): i) summary outputs and ii) detailed outputs. A summary output allows the user to rapidly review the landscape of sequence uniqueness across a region of interest. Further, we provide a visual summary plot directly adjacent to the table. Detailed output shows the locations of matched sequences and neighboring sequences with positions of mismatched nucleotides. To enable efficient online querying, the detailed output displays only the first 1000 lines. To allow the user to obtain results for queries that extend beyond 1000 lines, files are written to an AWS S3 bucket with a download link generated for users which is available for one hour.

### Data structure of KmerKeys

**Figure 2.**
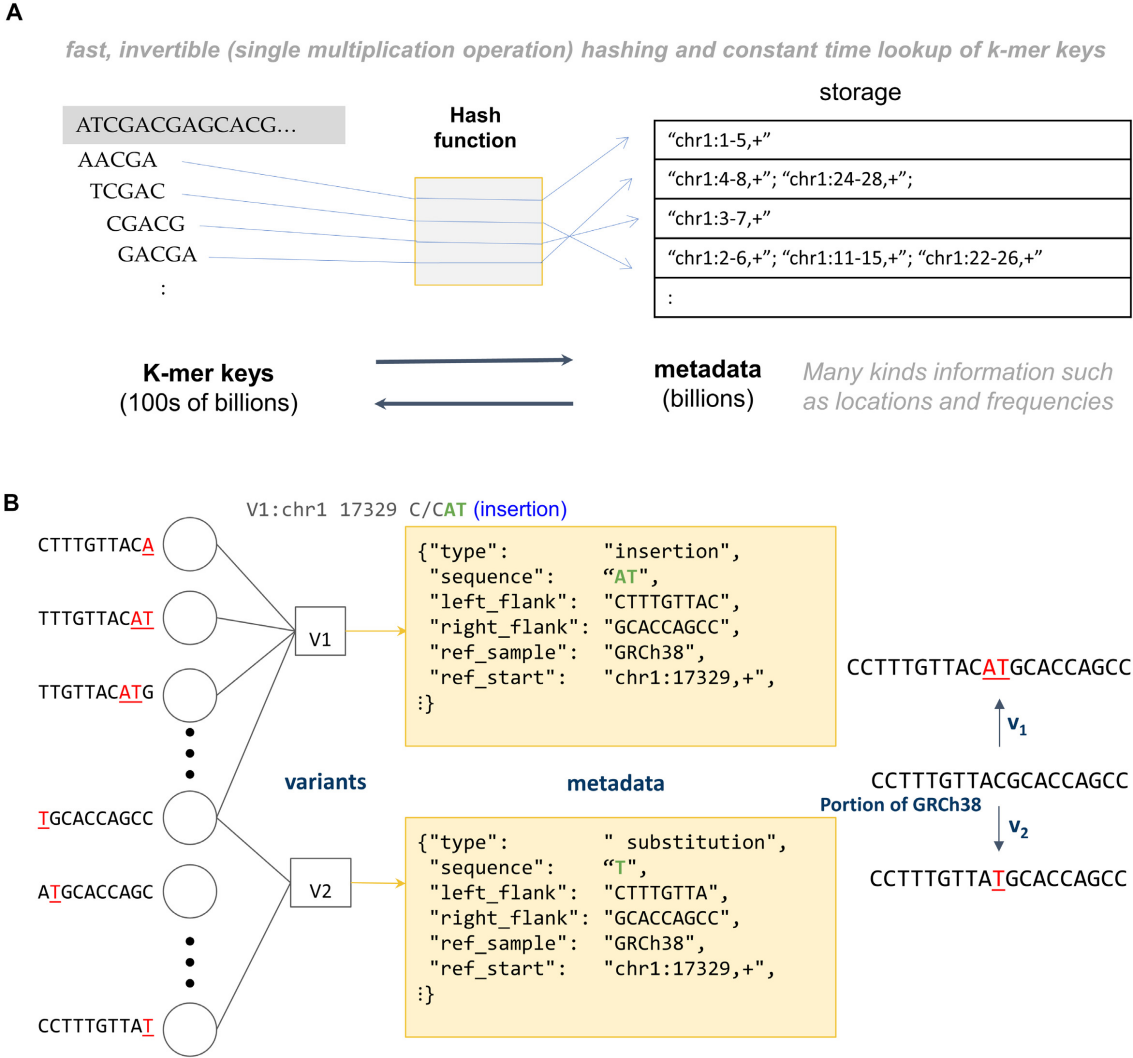
Overview of KmerKeys. (**A**) Data structure of KmerKeys. The hash function associates each k-mer key (sequence) with its metadata (locations and frequencies). (**B**) k-mer representation of variants. In the bipartite variant graph, each k-mer key (circle) is a k-mer generated by a variant in the GRCh38 sequence. The k-mer keys are associated with metadata (square), which includes the variant coordinate, sequence, type, and other useful information.

KmerKeys is a performant data structure that associates arbitrary genomic metadata with k-mer keys, allowing for large query speed and fuzzy search. In the hash table of KmerKeys, the bipartite variant graph has billions of k-mers (circles) and millions of locations (squares) (Figure 2A). For GRCh38 and the CHM13 assembly, KmerKeys has the hash table of all k-mers from both genomes as keys and associates them with the following metadata: i) the frequencies of the k-mer in GRCh38/CHM13 assembly and ii) the k-mer location(s) in GRCh38/CHM13 assembly. Therefore, each location will be associated with a given short sequence at that position, but also could be linked to multiple locations if the k-mer appears multiple times. To increase the performance, we employed a number of mathematical concepts previously unexploited in the k-mer indexing setting. They include an invertible Fibonacci hash function together with linear hash collision resolution and a quotient filter-inspired bitpacking scheme. Together, these features offer fast (constant expected time) queries that leverage memory caching for speed, bitpacking to reduce space and a simpler implementation than related data structures like the quotient filter (see Supplementary Methods). Further, we used this hash table to associate k-mers with metadata, thereby supporting optional memory mapping of values (the metadata) or the keys to reduce memory usage, and optimizing further for the setting of indexing locations and counts in a FASTA file. As a result, our implementation of this hash table supports millions of table lookups per second on a single thread. Basically, KmerKeys is designed to provide fast queries, O(1), or constant time in k and in the length of the indexed sequence, at the expense of extra memory relative to existing indexing tools including Burrows-Wheeler Transform (BWT) search. The scaling of BWT is logarithmic, O(m log n), in the indexed sequence length while ours is constant, O(1) for a single search and O(m) for multiple searches, but independent of n, which is the indexed sequence length.

### K-mer based representation of variants

We developed a method to represent genetic variation that includes single nucleotide variants **(SNVs)** and insertion deletions **(indels)**. Basically, the set of k-mers for a given length k overlapping the substituted base pair or spanning the insertion or deletion were associated with the coordinates based on GRCh38 (Figure 2B). For a single base pair substitution, this is the set of k-mers overlapping the substituted base pair. For short indels, this is the set of k-mers spanning the insertion or deletion (see Supplementary Methods). This representation of a variant allows use of the same schema that we used for indexing assemblies; a collection of variants represented in this way corresponds to a bipartite graph, with k-mers on one side and variants on the other denoted as circles and squares in Figure 2B. Importantly, this representation of a variant does not depend on a reference coordinate system. Therefore, we can associate any assembly coordinates with any other kind of metadata, like clinical information.

### Web implementation

We developed KmerKeys in the Julia programming language (17,18). The primary benefit of Julia is its level of language expressiveness and concision similar to Python, enabling rapid prototyping and experimentation without sacrificing much performance relative to compiled languages like C and C++. Thus, using Julia enabled us to prototype and release a performant version of our tool in the same language, which accelerated development. The front-end is a web app created using Angular (https://angularjs.org/). The front-end interfaces with a computational back-end running on a separate server, an AWS EC2 instance with sufficiently large memory to support billions of k-mer indices. Queries submitted via the front-end are sent to the back-end, which generates a response returned via the front-end.

## RESULTS

### Uniqueness of k-mers in the human reference genome (GRCh38) and the T2T assembly

**Figure 3.**
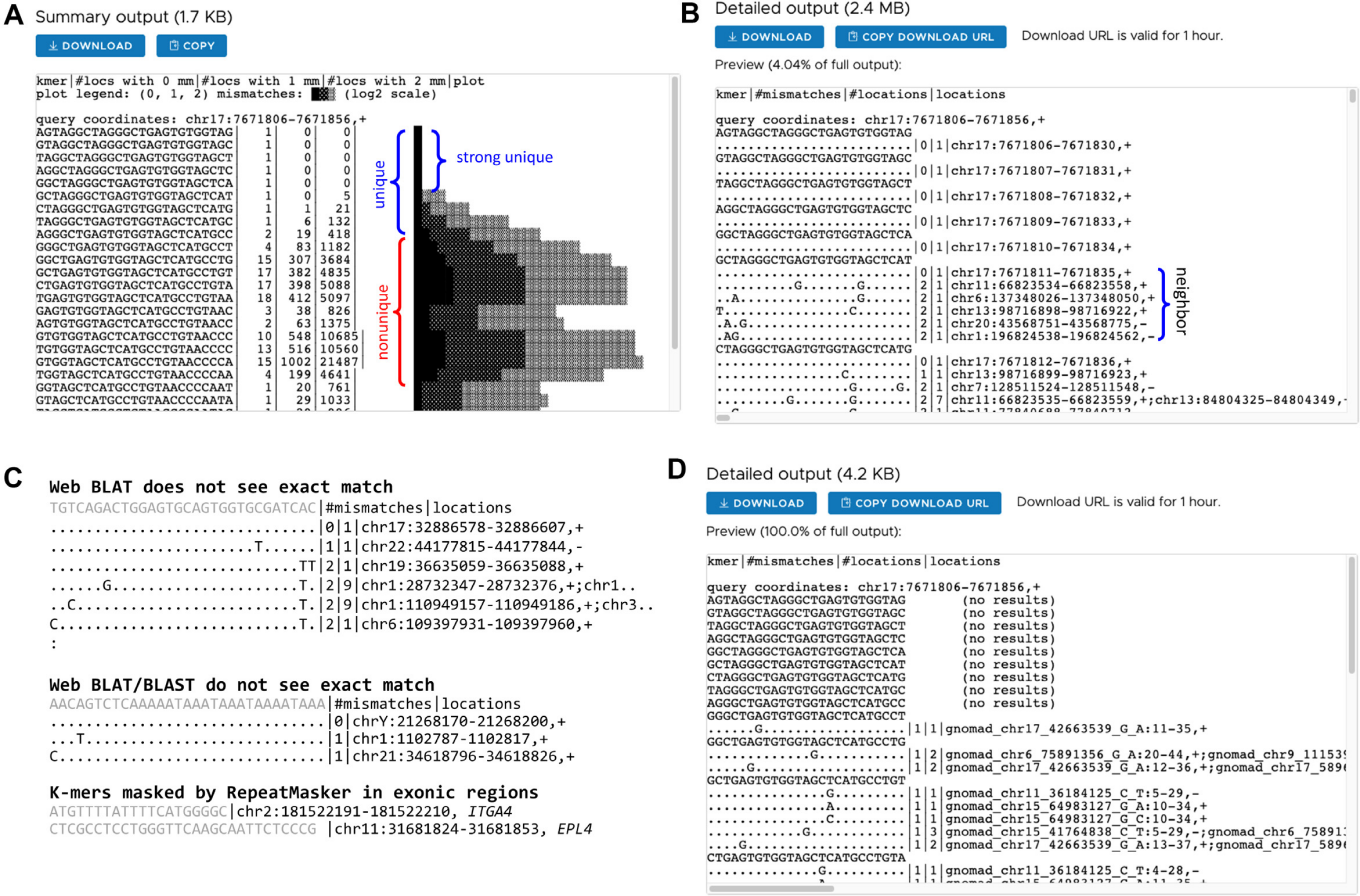
The uniqueness of k-mers measured by fuzzy search. (**A**) Example of summary output. The summary output displays each k-mer sequence in sequential order with counts of k-mers at each edit distance up to 2 mismatches, along with a plot that visually displays those counts. (**B**) Example of detailed output. Identical nucleotides are indicated by a dot (.) and different nucleotides are shown at their positions. The example search result showed that the k-mer sequences from the first 5 positions are unique within 2 mismatches while the sequence at the 6th position has 5 neighbor sequences with 2 mismatches. For instance, the 25-mer at the 6th position is identical to the 25-mer at chr6:137348026–137348050 except for two mismatches: i) A instead of T at the 3rd base position and ii) G instead of A at the 19th base position. (**C**) Example k-mers with unique exact matches identified by KmerKeys but not found by the web versions of BLAT/BLAST. (**D**) Example of detailed output from a KmerKeys web application query of 25-mers in gnomAD v2 in an intronic region of TP53.

Knowing the uniqueness of any sequence is critical information for a range of applications. This property is characterized by outputs of KmerKeys. Figure 3A and B show the summary and detailed outputs from the query of the coordinates, chr17:7671806–7671856 of GRCh38, which is located within the intron between exons 5 and 6 of *TP53*. The example search result showed that the k-mer sequences from the first 5 positions are strongly unique within 2 mismatches while the sequence at the 6th position has 5 neighbor sequences within 2 mismatches (Figure 3A). Detailed output shows the nucleotides and position of mismatches relative to query k-mers (Figure 3B). Similar trends were observed in the CHM13 assembly although there are minor differences. As shown in Supplementary Figure S1, the 9^th^ 25-mer is not unique in GRCh38 but is unique in the CHM13 assembly. These examples demonstrated that fuzzy, approximate searching provides the extent of uniqueness of k-mers. The information about neighbor k-mers is not easily retrieved by widely used tools such as Jellyfish (11) and KMC (12).

In addition, KmerKeys offers accurate search capabilities for specific short sequence that are an improvement over existing tools such as BLAST (19) and BLAT (20). For example, KmerKeys identified k-mers appearing uniquely in GRCh38 which were not identified by BLAST or BLAT (Figure 3C). We randomly sampled 100,000 unique 20, 21, 30 and 31-mers in GRCh38 (that is, each k-mer occurs at exactly one position in GRCh38). BLAT failed to identify approximately 1% of 20 and 21-mers and about 0.4% of 30 and 31-mers (Supplementary File S1). We also found that the web-based BLAST (21), though not the standalone software, sometimes missed unique k-mers (Supplementary Figure S2). In general, these missed unique k-mers contain the over-represented (appears more than 1024 times) 11-mers and the vast majority of them would be masked as repeat elements by RepeatMasker. Interestingly, several of the missing k-mers were located in coding regions (Figure 3C). To save computation time, BLAT and BLAST utilize 11-mers for the initial search for potential genomic regions where the actual sequence could be found. KmerKeys, on the other hand, simply indexes all k-mers from a given reference, thus guaranteeing the comprehensive searches.

### Population variant searching from gnomAD

We demonstrated the extensibility of our data structure for population-based genetic variation. This involved generating an index of all exonic variants in gnomAD (v2.1.1) through our k-mer-based variant representation. KmerKeys linked 17,119,203 variants in gnomAD with 523,498,431 31-mers. Users can query whether a sequence or coordinates based on GRCh38 contains the variants reported in gnomAD. We demonstrate an example using the gnomAD variants found in a genomic region of *TP53*, (Figure 3D). All the 25-mers within this region except 10 bp of the upstream portion overlap with at least one variant. It is important to note that none of the k-mers associated with variants are present in GRCh38. The fuzzy search function makes it possible to demonstrate how variants with unique k-mers from other genomes can be mapped back to the reference. This feature is unique among web-based resources. This function could provide useful information about whether 20-mers of interest could be unique in other individuals. In addition, we provide the compressed bed file (Supplementary File S2) that contains all indexed variants with their 21 different allele frequencies (AFs). Users can download it and quickly retrieve all 21 AFs of variants based on the GRCh38 genomic coordinates using tabix of Samtools (22). In fact, we designed primers for *RPP30*, a typical control gene for human DNA, that bind to genomic regions where no variants are reported by gnomAD (23). This feature enables to maximize the on-target rate for primers.

## DISCUSSION

In this study, we describe KmerKeys, a web data application that provides k-mer-based querying of human genome assemblies. For this application, we achieved the following: 1) we developed a data structure that efficiently and accurately associates arbitrary metadata with k-mers, 2) we devised a k-mer-based representation of variants that allows lists of variants to be jointly indexed with assemblies and primary sequencing, and 3) we launched a web application demonstrating the above, allowing users to query the locations and counts of k-mers in two whole human genome assemblies and exonic gnomAD v2 variants. KmerKeys has the potential to be used for DNA primer design and CRISPR/Cas9 target design. Using its search function, one can identify primer candidates that have the potential for off-target sites. Further, our data structure could provide a framework for representing variants at the population level and across multiple genomes simultaneously.

## DATA AVAILABILITY

KmerKeys is freely accessible at https://kmerkeys.dgi-stanford.org.

## Supplementary Material

gkac266_Supplemental_File
