# Supplementary material for: KmerKeys: a web resource for searching indexed genome assemblies and variants
Source: Nucleic Acids Res. 2022 Apr 26;50(W1):W448–53. doi: 10.1093/nar/gkac266 (PMC9252721; doi:10.1093/nar/gkac266)
Supplement: gkac266_Supplemental_File [file gkac266_supplemental_file.pdf]

SUPPLEMENTARY METHODS AND FILE

KmerKeys: a web resource for searching indexed genome assemblies and variants

Dmitri S. Pavlichin[1,†], HoJoon Lee[1,†], Stephanie U. Greer[1], Susan M. Grimes[2], Tsachy Weissman[3] and Hanlee P. Ji[1,2,*]

[1] Division of Oncology, Department of Medicine, Stanford University School of Medicine, Stanford, CA, 94305, United States

[2] Stanford Genome Technology Center West, Stanford University, Palo Alto, CA, 94304, United States

[3] Department of Electrical Engineering, Stanford University, Palo Alto, CA, 94304, United States

[†] Co-first authors

* Corresponding author

Hanlee P. Ji

Division of Oncology, Department of Medicine – Stanford University School of Medicine

CCSR 1115, 269 Campus Drive

Stanford, CA 94305-5151

Email: genomics_ji@stanford.edu

Tel: 650-721-1503

Fax: 650-725-1420

SUPPLEMENTARY FILE1: this file is available at

https://dna-discovery.stanford.edu/publicmaterial/web-resources/KmerKeys/

SUPPLEMENTARY FILE2: this file is available at

https://dna-discovery.stanford.edu/publicmaterial/web-resources/KmerKeys/

SUPPLEMENTARY METHODS

1. Hash table

Our hash table T is parametrized by integer k (k-mer length), integer A (alphabet size; A=4 for the 4 nucleotides {A,C,G,T}), integer N (the number of slots in an array), and integer H, the maximum distance of a key from its “home slot” (defined below). Keys farther than H slots from their home slot are stored in an overflow table T_{ov}. Additionally, we add trait-like parameters allowing for different behaviors of our data structure: Boolean IsSorted (controls whether keys are stored in a sorted order) and Boolean AllowDuplicates (controls whether duplicate keys are allowed).

- Integer k: k-mer length
- Alphabet size A (A = 4 for the nucleotides {A,C,G,T})
- Integer H (≥ 0): the maximum distance of a key from its “home address” (defined below)
- Integer N (≥ H): the number of slots in an array of slots

Keys farther than H slots from their home slot are stored in an overflow table T_{ov}. Additionally, we add trait-like parameters allowing for different behaviors of our data structure:

- Boolean IsSorted – controls whether keys are stored in a sorted order
- Boolean AllowDuplicates – controls whether duplicate keys are allowed

Several parameters are derived from the parameter choices above, and are convenient to reference below:

- A pair of integers U and V such that $U * V = 1 \pmod{A^k}$ and U is the nearest integer to A^k / ϕ with no prime factors in common with A^k, where ϕ is the golden ratio: multiplication by U (resp. V) hashes (resp. unhashes) k-mer keys.
- Integer $L = \lceil A^k / (N - H) \rceil$, where $\lceil . \rceil$ denotes the ceiling function: L is the number of distinct k-mers with the same "home address" (defined below).
- Integer $B \geq \lceil \log_2(H * L + 1) \rceil$: the number of bits per slot in the hash table. In our implementation we round B up to a multiple of 8, so that a slot is some integer number of bytes.

The N slots of hash table T are addressed by address q in {0,1,…,N-1}. Let T[q] denote the numerical value stored at address q in T. Since B bits are used per slot, then T[q] is in $\{0,1,\ldots,2^B-1\}$.

Let x be a k-mer and let underlines denote hashed quantities, e.g. $\underline{x} = U * x \pmod{A^k}$ and $x = V * \underline{x} \pmod{A^k}$.

The slot at address q is either "empty" or corresponds to a hashed k-mer $\underline{x}_q$ (and unhashed k-mer x_q). Slot q is empty if T[q] = 0. If slot q is not empty (if T[q] ≠ 0) , then the corresponding stored (hashed) k-mer is computed as:

$$\underline{x}_q = q * L + (L - T[q])$$

The "home address" of (hashed) k-mer $\underline{x}$ is the unique non-negative integer q satisfying the equation:

$$\underline{x} = q * L + r$$

with r in {0,1,…,L-1}. That is, q is the quotient and r the remainder upon division of $\underline{x}$ by L.

Matching up the previous two equations, we see that if k-mer $\underline{x}$ is stored in its home slot q, then the value at address q is T[q] = L – r in the set {1,2,…,L}.

2. Inserting and looking up keys

Given a k-mer x and table T, first hash x to obtain $\underline{x}$, then compute the home slot q and remainder r, and then find the nearest available slot, scanning slot addresses sequentially upwards starting from the home address q. That is, find the smallest integer h (≥ 0) such that either $\underline{x}_{q+h}$ == $\underline{x}$ or slot q + h is empty. In the first case, $\underline{x}$ is already in the table and there is nothing to do (unless we are also tracking the count of each k-mer; see [section] below). In the second case, if h ≤ H (the maximum allowed displacement from the home address), then write T[q+h] ← h * L – r to address q + h. If h > H, then signal that an overflow occurred, and write x to overflow table T_{ov}.

Pseudocode for inserting an element:

Inputs: hash table T, k-mer x
Outputs: 1) address at which x was inserted into T, 2) a Boolean signal denoting whether overflow occurred (i.e. too many hash collisions occurred)

```
def insertkey(T, x):
    x ← U * x (mod |X|^k)          # hash x
    q ← div(x, L)                   # quotient upon division by L
    r ← rem(x, L)                   # remainder upon division by L
    for h in 1 to H:                # at most H hash collisions
        if T[q] == 0:               # if slot is empty
            T[q] ← h * L - r        # write key to table
            return (q, false)       # no overflow
        else:                       # slot is occupied
            y ← (q + 1) * L - T[q] # hashed k-mer stored in slot
            if y == x:              # key is already in the hash table
                return (q, false)  # no overflow
        q ← q + 1                   # move to next slot
    return (q, true)                # overflow occurred
```

If an overflow occurs, then x is stored in an overflow table.

3. Reading an element from the table

Inputs: hash table T, k-mer x

Outputs: 1) address at which x is in T, 2) a Boolean signal denoting whether x is in T (output 1 is undefined if this signal is false), 3) a signal denoting whether overflow occurred (i.e. too many hash collisions occurred)

```
def locatekey(T, x):
    x ← U * x (mod |X|^k)             # hash x
    q ← div(x, L)                      # quotient upon division by L
    r ← rem(x, L)                      # remainder upon division by L
    for h in 1 to H:                   # at most H hash collisions
        if T[q] == 0:                  # if slot is empty
            return (q, false, false)   # x not in table
        else:                          # slot is occupied
            y ← (q + 1) * L - T[q]     # hashed k-mer stored in slot
            if y == x:                 # key is already in the hash table
                return (q, true, false) # no overflow
         q ← q + 1                     # move to next slot
    return (q, false, true)            # overflow occurred
```

If an overflow occurs, then check the overflow table for x.

4. K-mer based representation of variants

As an example, consider a particular variant: chr1:17329 C/CAT -- an insertion of the novel sequence AT following the 'C' at position chr1:17329 in GRCh38. Then the reference sequence in GRCh38, lacking the variant, is:

```
...CCTTTGTTACGCACCAGCC...
```

And a "patched" version instead containing the alternate allele is:

```
... CCTTTGTTACATGCACCAGCC...
```

where the inserted sequence is underlined. This set of novel k-mers overlaps any part of the variant allele, as shown in **Figure 2B**. Variant metadata can also include the variant coordinates in the reference genome as well as adjacent k-mers that are not affected by the variant, labeled "left_flank" and "right_flank" in **Figure 2B**. These extra metadata features make it straightforward to reconstruct a VCF-style representation of a variant from its graphical representation, and to map the variant to new assemblies. By associating k-mers with variants, we enable fuzzy sequence-based search of a collection of variants. This function has some novel properties. For example, with a VCF file one can query a set of

variants based on genome coordinates. However, it is less easy to do so based on the actual sequence containing the variation. Using a conventional approach, sequence-based variant querying requires one to first align the sequence to a reference genome and then use some other method to describe the set of variations. In contrast, our method permits associating short DNA sequences directly with k-mers and their metadata. A fuzzy search is achieved by querying all sequences within a given Hamming radius of a query sequence.

Fuzzy searching of k-mer-represented variants also enables robustness to the case of ambiguous variant definitions. For example, a substitution “AC/GG” may be represented as a pair of one-base-pair substitutions or a single two-base-pair substitution. By returning all approximately matching variants within some tolerance one can identify a maximum number of mismatches per k-mer.

Supplementary Figures

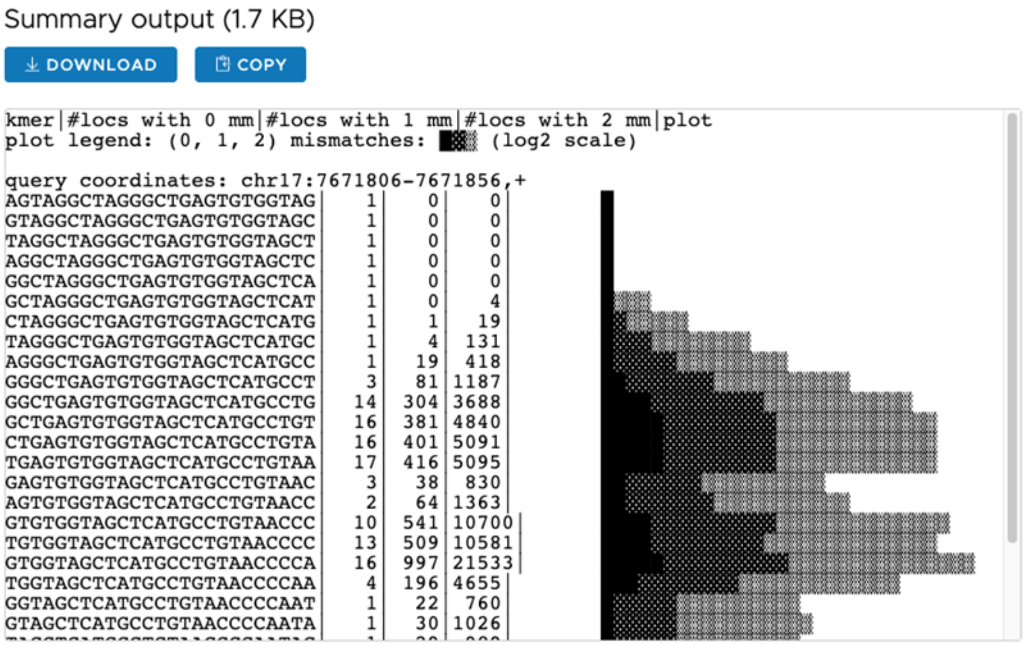

Supplementary Figure 1. The summary output of from the query of the coordinates, chr17:7671806-7671856 of CHM13.

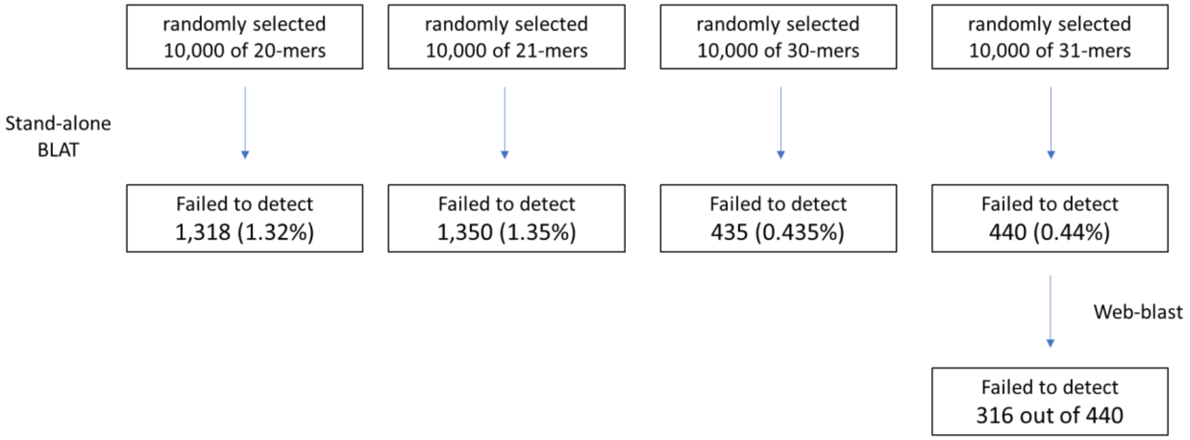

Supplementary Figure 2. Using stand-alone BLAT, we failed to identify 1,318 of the 20-mers (1.32%), 1,350 of the 21-mers (1.35%), 435 of the 30-mers (0.435%) and 440 of the 31-mers (0.44%), respectively. We also found that the web-based BLAST, though not the standalone software, sometimes misses unique k-mers. The same 100,000 randomly sampled unique 31-mers were identified by standalone BLAST, but web-based BLAST fails to find 316 of the uniquely occurring 31-mers among 440 that were not found by BLAT.
